# Chronotoxici‐Plate Containing Droplet‐Engineered Rhythmic Liver Organoids for Drug Toxicity Evaluation

**DOI:** 10.1002/advs.202305925

**Published:** 2024-05-08

**Authors:** Jiaqi Zhou, Yi‐chun Huang, Wanlong Wang, Jiawei Li, Yibo Hou, Ziqi Yi, Haowei Yang, Keer Hu, Yu Zhu, Zitian Wang, Shaohua Ma

**Affiliations:** ^1^ Tsinghua Shenzhen International Graduate School (SIGS) Tsinghua University Shenzhen 518055 China; ^2^ Tsinghua‐Berkeley Shenzhen Institute (TBSI) Shenzhen 518055 China; ^3^ Key Lab of Industrial Biocatalysis Ministry of Education Tsinghua University Beijing 100084 China

**Keywords:** chronotoxicity, Cry1, droplet‐engineered liver organoid, oxaliplatin

## Abstract

The circadian clock coordinates the daily rhythmicity of biological processes, and its dysregulation is associated with various human diseases. Despite the direct targeting of rhythmic genes by many prevalent and World Health Organization (WHO) essential drugs, traditional approaches can't satisfy the need of explore multi‐timepoint drug administration strategies across a wide range of drugs. Here, droplet‐engineered primary liver organoids (DPLOs) are generated with rhythmic characteristics in 4 days, and developed Chronotoxici‐plate as an in vitro high‐throughput automated rhythmic tool for chronotherapy assessment within 7 days. Cryptochrome 1 (*Cry1*) is identified as a rhythmic marker in DPLOs, providing insights for rapid assessment of organoid rhythmicity. Using oxaliplatin as a representative drug, time‐dependent variations are demonstrated in toxicity on the Chronotoxici‐plate, highlighting the importance of considering time‐dependent effects. Additionally, the role of chronobiology is underscored in primary organoid modeling. This study may provide tools for both precision chronotherapy and chronotoxicity in drug development by optimizing administration timing.

## Graphical abstract

A Chronotoxici‐plate containing droplet‐engineered rhythmic liver organoids has been developed for chronotherapy studies and chronotoxicity evaluation.

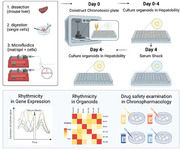

## 1 Introduction

The circadian clock orchestrates daily rhythms in human biochemical, physiological, and behavioral functions. Its disruption has been implicated in various human diseases, potentially contributing to metabolic syndrome. At the molecular level, the clock regulates rhythmicity in gene expression, cell division, and DNA repair, hinting at potential connections between circadian rhythms and both the pathogenesis and therapeutic interventions of cancer.^[^ ^1^ ^]^ As research progresses and understanding deepens, circadian rhythm research has transformed from a mere biological model into a crucial component of future clinical and pharmaceutical research. Emergencies that manifest at specific times of day, such as nocturnal asthma^[^ ^2^ ^]^ and glucose peak at dawn,^[^ ^3^ ^]^ are among the short list indications of circadian pathologies. Also, some studies demonstrated a higher incidence of heart attacks during morning hours and reduced risk associated with afternoon cardiovascular surgeries compared to those performed in the morning.^[^ ^4^ ^]^ These findings may be attributed to time‐dependent vulnerabilities linked to oscillations in blood pressure and heart rate,^[^ ^5^ ^]^ thromboembolic risk,^[^ ^6^ ^]^ arrhythmogenicity,^[^ ^7^ ^]^ and inflammation.^[^ ^8^ ^]^ While the detrimental effects of circadian rhythm disruption are well‐documented, the potential health benefits of harnessing or rectifying biological timing, including chronotherapy, remains underexplored.^[^ ^9^ ^]^ Balsalobre et al. successfully developed a circadian model that replicates the oscillatory expression of genes observed in vivo, by treating two‐dimensional (2D) cell line (rat‐1 fibroblasts or H35 hepatoma cells) with high concentrations of serum.^[^ ^10^ ^]^ The application of serum shock (SS) stimulated the expression of specific genes to mimic homeostasis in vivo.

Chronotherapy involves drug delivery at the appropriate phase of circadian rhythm to achieve optimal efficacy or minimal toxicity. And the rationale for this strategy is the performance of mechanism of action (MoA),^[^ ^11^ ^]^ pharmacokinetics and metabolism,^[^ ^12^ ^]^ as well as toxicity varying with time.^[^ ^13^ ^]^ The failure to consider chronotherapy can result in the overlooked potential of valuable drugs. A notable example is the initial high toxicity observed in clinical trials of oxaliplatin, where the benefits of chronotherapy were not considered.^[^ ^14^ ^]^ Synchronizing drug administration with the circadian rhythm, specifically at 16:00, has proven effective in enhancing therapeutic outcomes while mitigating drug toxicity. A significant portion of best‐selling and World Health Organization (WHO) essential drugs, including Rituxan, target the products of rhythmic genes.^[^ ^15^ ^]^ These drugs, often with short half‐lives, could benefit from timed dosage. Further, the need is pressing for researchers to incorporate circadian time in pre‐clinical and clinical phases of new drug development, as well as in evaluation of novel treatments. Applying chronotherapy to previously unsuccessful drugs, following the oxaliplatin example to align the administration time, could also yield promising results in the balance of therapeutic efficacy and drug toxicity. However, traditional approaches using model animals are hampered by complex procedures, high costs, and low throughput, hindering large‐scale drug screening and multi‐timepoint drug administration strategies.

Organoids are in vitro self‐assembling, three‐dimensional (3D) cellular structures that retain key characteristics of the respective tissues.^[^ ^16^ ^]^ They serve as a powerful tool for biomarker research, drug screening, and accurate prediction of therapeutic efficacy. When combined with high‐throughput assays, organoids can reduce the time and cost of experiments. Microfluidic droplets provide monodisperse and tailorable templating volumes for cellular compartments, where primary or pluripotent cells grow and develop into reproducible organoids, termed droplet‐engineered organoids (DEOs).^[^ ^17^ ^]^ This technology provides a high‐throughput, standardized and cost‐effective choice to conduct large‐scale drug screening with multi‐timepoint drug administration strategies.

As an emerging tool, multiple attempts have been made on organoid‐based circadian rhythm research.^[^ ^18^, ^19^, ^20^, ^21^, ^22^ ^]^ Stokes et al. assessed the circadian transcription rhythms in intestinal organoids and tested *Bmal1*‐dependent proliferation and self‐renewal.^[^ ^21^ ^]^ Rosselot et al. found circadian phase‐dependent necrotic cell death responses to Clostridium difficile toxin B (TcdB) in intestinal organoids.^[^ ^19^ ^]^ Notably, there is a significant gap in the literature regarding the establishment of fully in vitro rhythmic organoids tailored for precision cancer chronotherapy. Our work stands out as a promising avenue for introducing a novel therapeutic paradigm, moving beyond its role as a conventional drug sensitivity and toxicity assessment tool.

Here, leveraging a high‐throughput DEO fabrication tool, we utilized microfluidics and 3D printing technologies to construct a “Chronotoxici‐plate”: a 96‐well plate filled with rhythmically synchronized droplet‐engineered primary liver organoids (DPLOs). The system enabled DPLOs establishment within 4 days, identified Cryptochrome 1 (*Cry1)* as a potential companion diagnostic marker, and realized assessment of oxaliplatin toxicity for chronotherapy within one week. Further, it demonstrated the effect of rhythm synchronization in liver organoid growth, suggesting it as a new dimensional intervention to improve primary organoid culture.

## 2 Results and Discussion

### 2.1 Establishment of Chronotoxici‐Plate Derived from Mouse Liver

**Figure 1:**
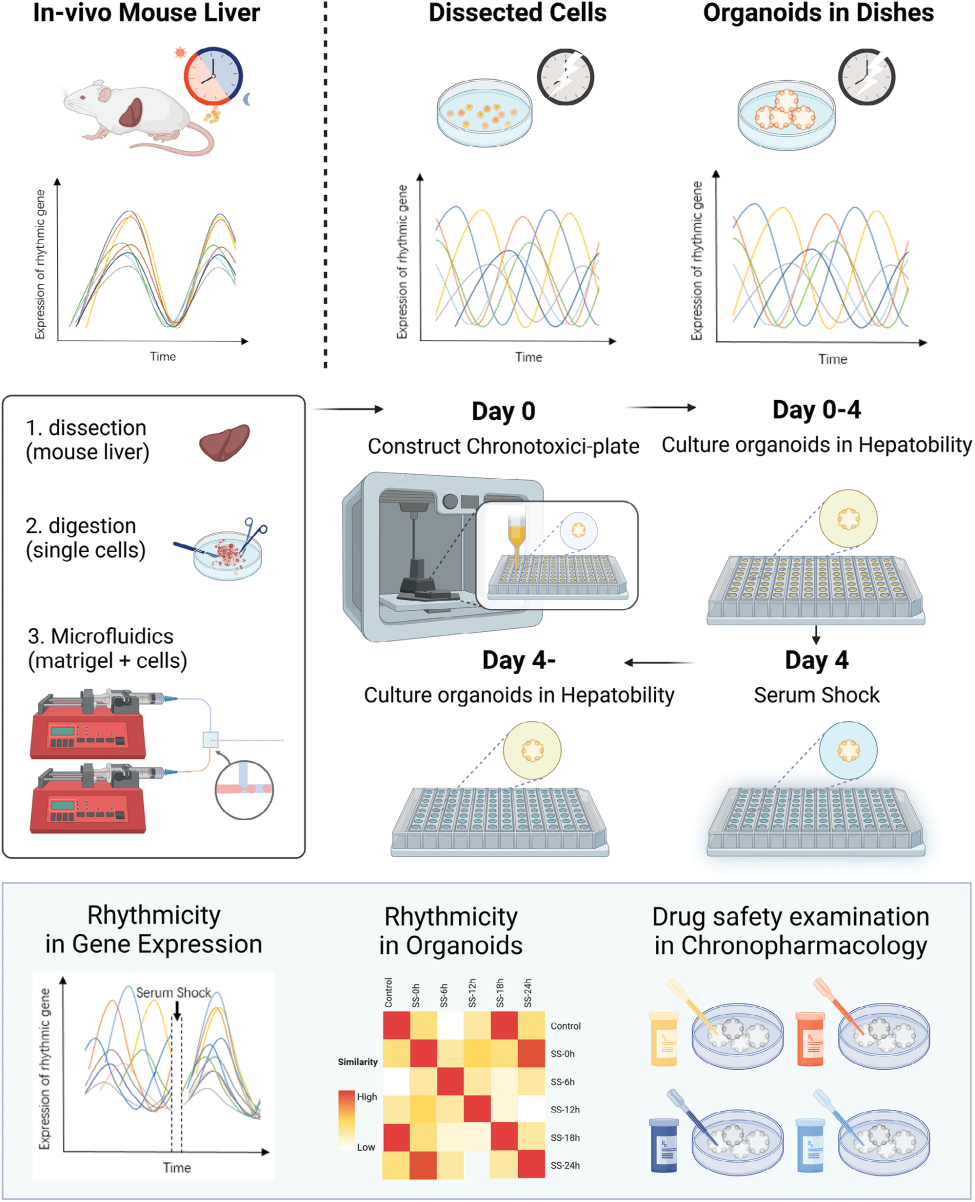
Schematic illustration of Chronotoci‐plate construction. The preservation of intrinsic tissue‐level circadian rhythms poses a challenge in isolated cells and primary organoids derived from these cells. To overcome this limitation, we designed the Chronotoxici‐plate: a formatted tool aimed at restoring rhythmicity for drug safety assessment. After four days of culture, the organoids within the plate are subjected to SS, then returned to their original environment, effectively synchronizing their cellular rhythms. The Chronotoxici‐plate represents a high‐throughput kit for the exploration of chronotoxicity, making it easier to study of drug responses in a time‐dependent manner.

Circadian biology is increasingly recognized as a crucial factor in enhancing drug efficacy and reducing toxicity.^[^ ^23^ ^]^ However, isolated cells exhibit poor synchronization of circadian cycles^[^ ^22^, ^24^ ^]^ and the large‐scale drug screening with multi‐timepoint drug administration is not satisfied. Therefore, there is currently a lack of rapid and efficient tools with circadian rhythmic features for drug screening. DEOs were formulated in a high‐throughput manner in a customized microfluidics setup, i.e., cascade‐tubing microfluidics (CTM). The organoids were individually bioprinted into a 96‐well plate and synchronized in rhythm on the 4^th^ day using SS, establishing the circadian drug screening organoids, i.e., DPLOs. We designated this organoid‐housed plate as “Chronotoxici‐plate” and employed it for subsequent drug screening purposes. *Cry1* was screened as a marker of chronotherapy. Using the screened gene as the drug safety assessment companion, we performed in‐depth analysis of rhythmic liver toxicity of oxaliplatin on the Chronotoxici‐plate (**Figure** **1**).

**Figure 2:**
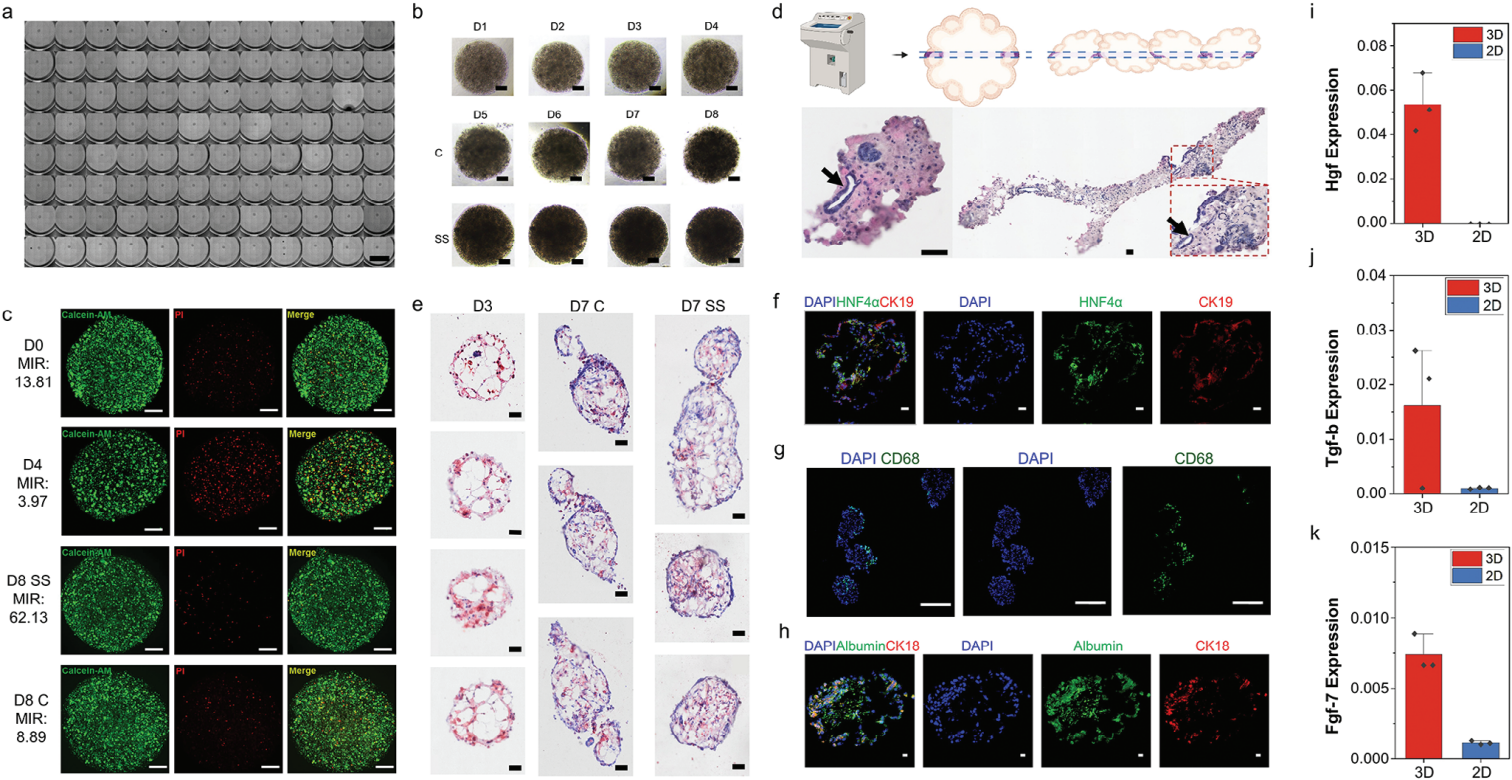
Cultivating DPLOs within the Chronotoxici‐plate. a) High‐content analysis of each well in Chronotoxici‐plate at Day 1. Every well in the 96‐plate contained a single DPLO. Scale bar = 500 µm. b) Observation of organoid culture process under bright‐field microscopy. DPLOs were subjected to SS or kept as control (C) after four days of culture, followed by continued cultivation until the eighth day (D8). Scale bar = 100 µm. c) Confocal microscopy image depicting the viability of DPLOs through whole mount staining using Calcein‐AM/PI live/dead staining. MIR: the mean green‐to‐red intensity ratio. Scale bar = 100 µm. d) Schematic representation and histological staining images of single DPLO and interconnected multi‐DPLOs at Day 4 after establishment. Scale bar = 80 µm. e) Oil Red O staining images of single DPLO and interconnected multi‐DPLOs at Day 3 and Day 7 with or without SS treatment. Scale bar = 50 µm. f, g, and h) Confocal image of a cryo‐sectioned DPLO co‐stained for HNF4a (green), CK19 (red), CD68 (green), albumin (green), CK18 (red), and DAPI (blue). Scale bar = 50 µm. i, j, and k) qRT‐PCR analysis of the expression levels of liver‐related growth factors in DPLOs and AML12 cell lines. i,k) * indicates p < 0.05.

To provide a fast and efficient tool, Chronotoxici‐plate, for circadian research and drug safety assessment, we reconstructed hepatobiliary microtissues in vitro^[^ ^25^, ^26^, ^27^, ^28^ ^]^ using primary cells isolated from healthy mouse liver^[^ ^29^ ^]^ to formulate DPLOs and patterned them in Chronotoxici‐plate (**Figure** **2**a). Each well contained only one DPLO. We utilized SS treatment at Day 4 to synchronize organoid cell phases, and the DPLOs were featured with higher cell density over time (Figure 2b). The results of the live/dead cell viability test indicated higher rates of cell survival at Day 8 for both the SS and control (C) groups, as shown by the mean green‐to‐red intensity ratios (MIR) (SS group, MIR_ss_ = 62.13; control group, MIR_c_ = 8.89, Figure 2c). The Adenosine triphosphate (ATP) luminescence assay revealed a gradual recovery of cell activity following an initial decline, at Days 1–4 and Days 5–6 respectively (Figure S1, Supporting Information). During the culture process, cell metabolic levels and viability rates first decreased from Day 1 to Day 4 possibly due to the harmful effect of primary cell extraction and adaption to the in‐vitro environment,^[^ ^30^, ^31^ ^]^ followed by noticeable cell proliferation from Day 4 to Day 8. This is in line with the scenario observed in classical primary liver organoids,^[^ ^32^ ^]^ or termed non‐engineered organoids^[^ ^17^ ^]^ (NEO), though the decline in ATP activity was greater and exhibited slower and considerably compromised recovery (Figure S2, Supporting Information).

Subsequently, we validated the structure and functionality of DPLOs. Hematoxylin‐eosin (HE) staining of the organoids after four‐day culturing revealed the presence of tubular structures (Figure 2d, black arrow) and densely packed cell clusters with organization (Figure 2d). Oil Red O staining revealed a noticeably higher cell density at Day 7 compared to Day 3 and a superior structural arrangement with no obvious *de novo* lipogenesis (Figure 2e).

Immunofluorescence (IF) analysis was conducted on the organoids at Day 4 demonstrated the presence of HNF4α+ hepatocyte clusters distributed within the organoids, co‐existing with a portion of CK19+ cholangiocytes (Figure 2f, Figure S3, Supporting Information). Additionally, Kupffer cells, characterized by CD68 expression, were also observed in the organoids (Figure 2g). Cytokeratin‐18 (CK18), an abundant intermediate filament protein in hepatocytes and cholangiocytes, was expressed in the tubular structures. Additionally, albumin, which reflects hepatocyte functionality and protein synthesis, exhibited widely expression in the organoids (Figure 2h; Figure S3, Supporting Information). The co‐expression of biliary and hepatocyte markers (CK19 and HNF4a), one of the characteristics of periportal hepatocytes of regenerating livers,^[^ ^33^ ^]^ has been validated as markers for functional liver organoids.^[^ ^29^, ^34^ ^]^ These findings demonstrate the success of structural development and functional maturation of DPLOs.

Organoid cultures have the potential to partially recapitulate tissue phenotypes and reproduce their paracrine activity compared to 2D cell cultures.^[^ ^35^ ^]^ The viability and preservation of in vivo‐like phenotypes in liver primary cells isolated from tissue have been shown to be challenging when cultured in a 2D environment (Figure S4, Supporting Information). This difficulty underscores the inherent limitations of traditional 2D culture systems in replicating the complex in vivo conditions essential for maintaining the functional characteristics of hepatic cells.^[^ ^36^, ^37^ ^]^ We also examined the expression of important paracrine factors involved in liver growth and development in DPLOs where hepatocytes isolated from the normal liver of a 3‐month‐old mouse, and AML12 (alpha mouse liver 12) cells of 2D culture after SS treatment. Our results revealed significantly higher expression levels of hepatocyte growth factor (Hgf),^[^ ^38^, ^39^, ^40^ ^]^ transforming growth factor beta (Tgf),^[^ ^41^, ^42^ ^]^ and fibroblast growth factor‐7 (Fgf‐7)^[^ ^43^, ^44^ ^]^ in DPLOs compared to the AML12 cells (Figure 2j–l) This highlights the advantage of droplet‐encapsulation‐derived organoids in reproducing tissue paracrine activity and their potential to simulate organ‐like features in vitro.

### 2.2 Rhythmicity Gaining in DPLOs in Chronotoxici‐Plate

**Figure 3:**
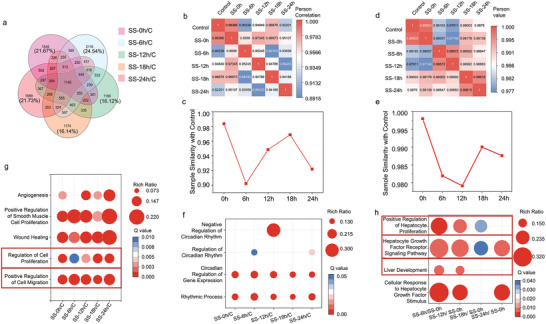
Synchronization of DPLOs via SS treatment. a) Venn diagram illustration of the overlap of differentially expressed genes (DEGs) among different groups from RNA sequencing. b) Sample Similarity Heatmap of clock‐controlled genes (CCGs) determined by RNA sequencing comparing samples from different time points after SS (SS‐0 h, SS‐6 h, SS‐12 h, SS‐18 h, SS‐24 h) with the control group. c) Temporal dynamics of CCGs expression from RNA sequencing in response to SS treatment compared to the control group. d) Sample similarity heatmap of gene expression profiles determined by mRNA sequencing across samples from various time points following SS compared to the control group. e) Temporal dynamics of gene expression profiles in response to SS treatment compared to the control group. f) Gene Ontology enrichment analysis of DEG sets by mRNA sequencing comparing SS DPLOs to control DPLOs. The analysis revealed significant enrichment of rhythmic‐related pathways. A threshold of Q‐value (corrected P‐value) ≤ 0.05 was applied, defining GO terms that exhibited significant enrichment among the candidate genes. The Rich ratio represents the ratio between the number of DEGs in a specific metabolic pathway and the total number of genes annotated to that pathway. Higher values indicate a greater degree of enrichment within the pathway. g and h) Gene Ontology enrichment analysis of DEG sets by mRNA sequencing comparing SS DPLOs to control DPLOs or the initial time point after SS. The analysis revealed significant enrichment of liver development and cell proliferation. A threshold of Q‐value (corrected P‐value) ≤ 0.05 was applied, defining GO terms that exhibited significant enrichment among the candidate genes.

To validate the synchronization of cellular rhythms in DPLOs of Chronotoxici‐plate, we sampled organoids every 6 hours starting from the end of SS treatment.^[^ ^45^, ^46^, ^47^ ^]^ The organoids were labeled as SS‐0 h, SS‐6 h, SS‐12 h, SS‐18 h, and SS‐24 h. The control group organoids were also sampled from the same batch of DPLOs at 0 h but without SS treatment. RNA sequencing analysis revealed that gene expression levels in samples from different time points after SS were similar to the control group, i.e., the non‐SS‐treated group (Figure S5,S6, Supporting Information). It suggested that SS did not induce widespread sustainable upregulation or downregulation of genes. A comparative analysis was conducted between the SS‐treated groups at various time points and the control group (SS‐0 h/C, SS‐6 h/C, SS‐12 h/C, SS‐18 h/C, SS‐24 h/C). The analysis revealed 1145 common differentially expressed genes (DEGs). While DEGs uniquely expressed in a specific SS group were proposed as genes subject to circadian rhythm, as shown in **Figure** **3**a. To evaluate the rhythmicity, the sample similarity of well‐acknowledged 12 clock‐controlled genes^[^ ^48^ ^]^ (CCGs) was assessed in the five comparison groups (SS‐0 h/C, SS‐6 h/C, SS‐12 h/C, SS‐18 h/C, SS‐24 h/C) through gene expression profiling. The results revealed a oscillation pattern of initial decrease, subsequent increase, and final decrease from 0 to 24 hours (Figure 3b,c). A similar oscillation pattern was observed in the CCGs and the whole transcriptomic profiling from 6 to 24 hours (Figure 3d,e). Collectively, it was revealed that rhythmic gene expression was recapitulated in DPLOs of Chronotoxici‐plate.

### 2.3 Liver Development in Rhythmic DPLOs

To further investigate pathway alterations in synchronized DPLOs, gene ontology (GO) enrichment analysis was performed between different time points of the SS groups and the control group. Under the criteria of Q value < 0.05, enriched pathways related to circadian rhythms were identified, including negative regulation of circadian rhythm (GO:00 42754), regulation of circadian rhythm (GO:00 42752), circadian regulation of gene expression (GO:00 32922), and rhythmic process (GO:00 48511) (Figure 3f). Additionally, enrichment was observed in pathways associated with regulation of cell proliferation (GO:00 42127), positive regulation of cell migration (GO:00 30335), as well as positive regulation of hepatocyte proliferation (GO:2 000 347), hepatocyte growth factor receptor signaling pathway (GO:00 48012), and liver development (GO:0 001889), indicating enhancement of cell proliferation, migration, and potential liver development in DPLOs after synchronization (Figure 3g,h). Besides, the survival of DPLOs after synchronization surpassed the control group by Day 6 and showed a significantly higher cell viability rate at Day 8 (Figure 2b,c). It may suggest a positive effect of rhythmic synchronization on DPLOs in terms of liver development and survival.

### 2.4 *Cry1* is a Rhythmic Marker in DPLOs

**Figure 4:**
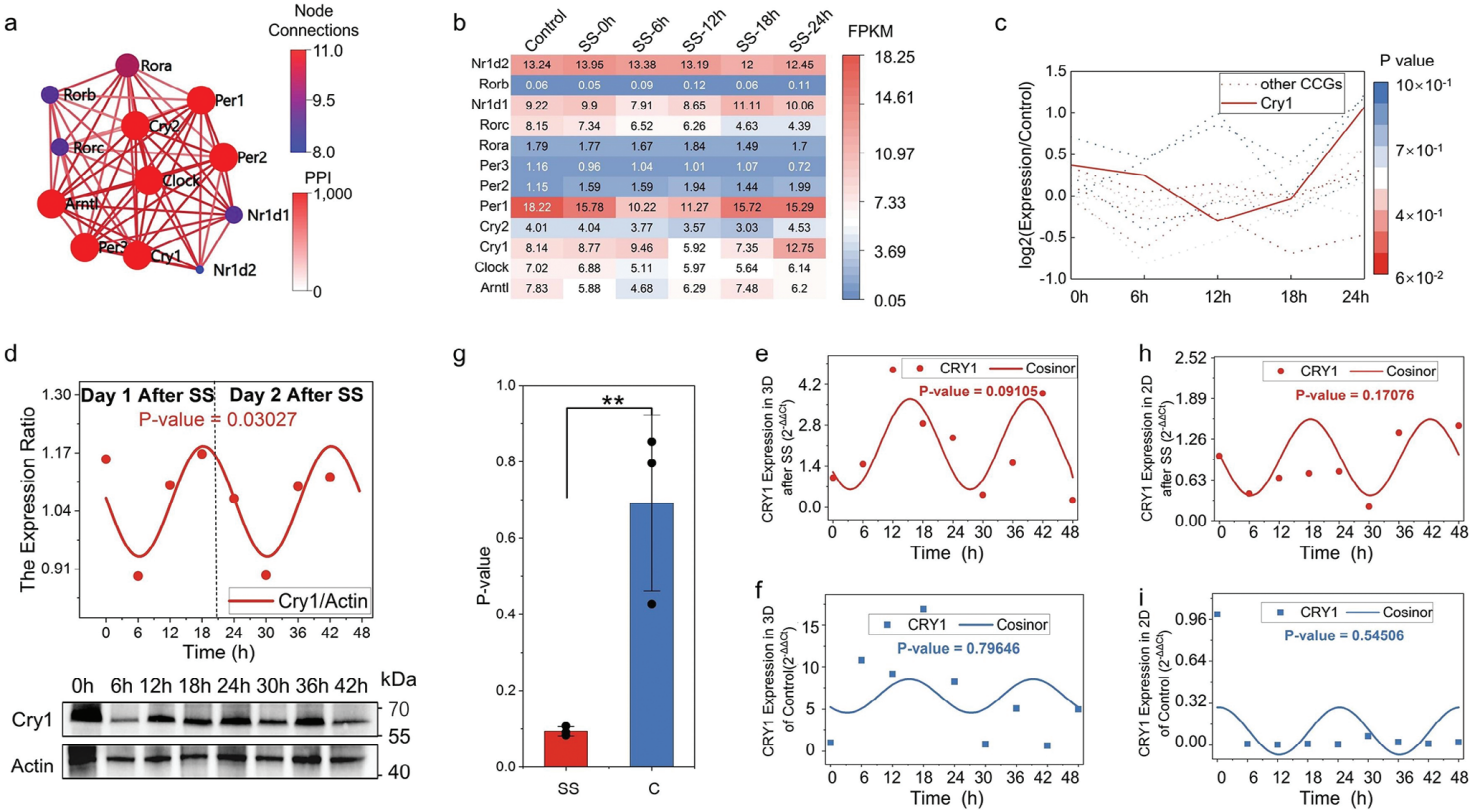
Identification of *Cry1* as a potential companion diagnostic indicator. a) Network representation of CCGs interactions using mRNA sequencing results. The color of the nodes indicates the number of connections, while the color of the edges reflects the degree of interaction. b) Heatmap of CCGs expression determined by mRNA sequencing comparing samples from different time points after SS with the control group. c) Fit of CCGs expression profiles at different time points after SS to a cosine function, and the p‐value was calculated.^[^ ^19^, ^22^, ^70^, ^71^, ^72^, ^73^ ^]^ d) Temporal dynamics of the ratio between *Cry1* and housekeeping gene Actin expression levels following SS treatment in western blot analysis. Each dot stands for the relative protein expression of *Cry1*, which were fitted to the cosine function, and the p‐value was calculated. e,f,h, and i) Fit of *Cry1* expression by qRT‐PCR analysis at different time points in DPLOs or AML12 cell lines with or without SS to a cosine function, and the p‐value was calculated. Each dot stands for the relative mRNA expression of *Cry1*. g) Comparison of the fit between the expression of *Cry1* gene, with or without SS, and a sine function. ∗∗ indicates p < 0.01.

To simplify and expedite the assessment of rhythmicity in DPLOs of the Chronotoxici‐plate, we identified the rhythmic marker in DPLOs. Based on the rhythmic expression of 12 CCGs observed in DPLOs (Figure 3b,c), protein‐protein interaction networks (PPI) analysis revealed close interactions among all CCGs, particularly the close interactions between *Cry1* and other genes (**Figure** **4**a). Analysis of CCG expression at different time points after synchronization showed *Cry1* expression oscillated around the expression values in the control group and exhibited the best fit to the periodic function (p‐value = 0.06158, Figure 4b,c).

Subsequently, we performed multiple replicates at the protein and mRNA levels to investigate the effects of synchronization on DPLOs of the SS group and the control group. At the protein expression level, Cry1 exhibited robust periodicity in expression ratio with the housekeeping gene Actin (p‐value = 0.03027, Figure 4d). At the mRNA expression level, following synchronization, DPLOs showed a lower p‐value for Cry1 gene expression fitting the sine function (p‐value = 0.09105, Figure 4e) compared to the control group (p‐value = 0.79646, Figure 4f). Multiple repeated experiments consistently demonstrated a significant effect of SS in reducing the p‐value for *Cry1* expression and sine function fitting in DPLOs (Figure 4g). This demonstrated *Cry1* emerges as a representative rhythmic marker in DPLOs. In contrast, SS in AML12 cells cultured in 2D showed a larger p‐value (p‐value = 0.17076) for *Cry1* expression fitting the sine function (Figure 4h,i), indicating a less pronounced effect of SS‐induced synchronization on 2D cells than on DPLO cells. This discrepancy may be attributed to the activation of numerous rhythmic‐related pathways (Figure 3f–h) and paracrine expression (Figure 2i–k) of 3D culture following SS treatment. These results underscore the importance of droplet‐encapsulation in generating rhythmically synchronized organoids.

### 2.5 Chronotoxici‐Plate is a Circadian Tool for Drug Toxicity Assessment

**Figure 5:**
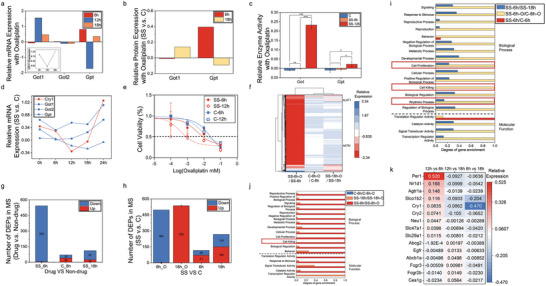
Drug safety assessment in Chronotoxici‐plate. a) qRT‐PCR analysis of liver injury‐related genes Got1, Got2, and Gpt, as well as the circadian rhythm gene *Cry1* expression level, in DPLOs following SS treatment or keeping as a control at different time points with a period of 36‐hour drug exposure. b) iTRAQ analysis of Got1 and Gpt in DPLOs following SS treatment at different time points with a 36‐hour drug exposure period. c) Differences in GOT and GPT enzyme activities in DPLOs with or without SS treatment following a 36‐hour exposure to drug or blank conditions. d) Relative expression of Got1, Got2, Gpt, and Cry1 by mRNA sequencing comparing samples from different time points after SS (SS‐0 h, SS‐6 h, SS‐12 h, SS‐18 h, SS‐24 h) with the control group. e) IC50 comparison of oxaliplatin applied on DPLOs treated with or without SS at different time points for a 36‐hour period. f) Protein profile heatmap of oxaliplatin or non‐oxaliplatin applied on DPLOs at 6 hours or 18 hours after SS group or control group. g and h) Comparison of DEGs by protein quantitation analysis in DPLOs treated with or without oxaliplatin over a 36‐hour period beginning at 6 hours or 18 hours after SS, as well as in the control group. i and j) KEGG pathway enrichment analysis of DEG sets by protein expression comparing oxaliplatin treatment and serum treatment DPLOs to control DPLOs. A threshold of Q‐value (corrected P‐value) ≤ 0.05 was applied, defining KEGG pathway that exhibited significant enrichment among the candidate genes. k) Temporal dynamics of drug target gene expression related to circadian rhythm and liver function for the top 100 best‐selling drugs.^[^ ^15^ ^]^ * indicates p < 0.05, **indicates p < 0.01, ***indicates p < 0.001.

Oxaliplatin, as a typical example of chronotoxicity, was marred by excessive toxicity in initial clinical trials, but subsequent successful chronotherapy investigations have propelled oxaliplatin to become one of the primary therapeutic options for cancer on a global scale.^[^ ^49^ ^]^ To evaluate the effect of rhythmic DPLOs in Chronotoxici‐plate on drug safety assessment, we treated Chronotoxici‐plate with oxaliplatin for 36 hours at different time points following synchronization. Glutamic‐oxaloacetic transaminase (GOT) and glutamic‐pyruvic transaminase (GPT) isoenzymes are known as crucial markers for assessing liver function.^[^ ^50^, ^51^ ^]^ Therefore, we measured the mRNA expression levels of cytosolic glutamic‐oxaloacetic transaminase‐1 (Got1), mitochondrial glutamic‐oxaloacetic transaminase‐2 (Got2), glutamic‐pyruvic transaminase (Gpt), along with the companion of the representative circadian gene Cry1. We assessed the expression of the rhythmic marker *Cry1* at the time points of drug delivery (6 h, 12 h, 18 h), which showed the pattern of an initial decrease followed by an increase (**Figure** **5**a). With the oscillated expression of rhythmic marker *Cry1* in varied time‐point after synchronization, the results revealed a slightly lower expression of GOT1 in SS‐6h‐O (the group treated with oxaliplatin for 36 hours at 6 hours after SS synchronization) followed by a higher expression in SS‐12h‐O and SS‐18h‐O, compared to the non‐SS‐treated group. GOT2 showed a slightly higher expression initially and then a slightly lower expression compared to the non‐SS‐treated group, with only minor differences observed. The expression level of GPT initially exceeded and showed a significant decrease in 12 h, followed by a slight increase above 18 h (Figure 5a). Proteomic analysis of DPLOs (Figure S7, Supporting Information) after oxaliplatin treatment beginning at 6, 12, and 18 hours after SS synchronization displayed similar trends for GOT1 and GPT as observed in the mRNA analysis (Figure 5b), as well as enzyme activity assays (Figure 5c). Furthermore, significant differences in enzyme activity indicative of liver damage were observed at SS‐6 h and SS‐12 h, which highlighted distinct impacts of drug administration at different time points after SS treatment on DPLOs (Figure 5c). In comparison to the non‐SS treated group, the SS‐12 h group exhibited notably higher GOT and GPT activities, suggesting a potential heightened sensitivity to the drug.

In RNA‐seq analysis, compared to the control group, the synchronized DPLOs without drug treatment displayed rhythmic changes in *Cry1* expression, but no corresponding rhythmic changes were shown in Got1, Got2 and Gpt (Figure 5d). It suggested that Got1, Got2 and Gpt did not have intrinsic rhythmicity. Furthermore, proteomic analysis of drug‐treated DPLOs revealed that the expression levels of GOT and GPT in the control group were intermediate between those in the SS‐6 h and SS‐18 h DPLOs (Figure S8, Supporting Information). Thus, the varying expression of GOT and GPT was induced by the different timing of oxaliplatin administration rather than SS itself.

Subsequently, cell viability assay was conducted to assess the drug safety. The results showed no significant differences in cell viability changes at different time points in the non‐synchronized groups (C‐6 h, C‐12 h). However, the rhythmic groups displayed substantial variations in cell viability after drug administration at different time points (SS‐6 h, SS‐12 h). Specifically, SS‐6 h DPLOs exhibited lower sensitivity to oxaliplatin compared to the SS‐12 h DPLOs. Compared to the non‐synchronization group, the SS‐12 h groups demonstrated significantly lower half maximal inhibitory concentration (IC_50_) values, indicating their higher sensitivity to the drug, while SS‐6 h DPLOs exhibited similar sensitivity to the drug as the non‐synchronization group (Figure 5e). AM‐PI staining yielded consistent results with the IC50 curves at an oxaliplatin concentration of 10^−4^ mM (Figure S9, Supporting Information). Together, it proved the liver toxicity of oxaliplatin was rhythm‐dependent, and SS‐DPLOs possessed the ability to evaluate the rhythm‐dependent toxicity.

Taking count of the expression of rhythmic marker *Cry1*, the sensitivity of DPLOs on oxaliplatin is in negative correlation with the expression of *Cry1* (Figure 5a). The results shed light on the potential of *Cry1* as a valuable marker for determining the optimal drug administration timepoint with minimized toxicity in clinical therapy.

### 2.6 Mechanisms of Varying Toxicity of Oxaliplatin on Chronotoxici‐Plate

To further investigate the effects of multi‐timepoint drug administration on rhythmic DPLOs, we performed Isobaric Tags for Relative and Absolute Quantitation (iTRAQ) analysis on DPLO treated with oxaliplatin at different time points (Figure S7, Supporting Information). The results revealed significant differences in protein expression levels at the 6‐hour after synchronization oxaliplatin‐treated group (SS‐6h‐O) with the 18‐hour group (SS‐18h‐O), compared to their respective untreated groups (SS‐6 h, SS‐18 h). Besides, there were minimal differences observed between the oxaliplatin‐treated group without synchronization (C‐6h‐O) and the untreated group (C‐6 h) (Figure 5f; Figure S10, Supporting Information). These findings indicate that rhythmic DPLOs possess varying levels of toxicity at different timepoint, consistent with the IC50 conclusions (Figure 5e). When comparing differentially expressed proteins (DEPs) in DPLOs with or without oxaliplatin treatment and synchronization, it was observed that SS‐6h‐O (compared to SS‐6 h, 540 DEPs) had more DEPs than SS‐18h‐O (compared to SS‐18 h, 111 DEPs), and both were higher than the DEPs in C‐6h‐O compared to C‐6 h (Figure 5g). Additionally, the number of DEPs in SS‐6h‐O versus C‐6h‐O and SS‐18h‐O versus C‐18h‐O was significantly greater than in the corresponding untreated groups (Figure 5h). Thus, oxaliplatin treatment resulted in a greater number of DEPs in rhythmic DPLOs compared to the untreated groups (Figure S11, Supporting Information). Rhythmic DPLOs demonstrated enhanced sensitivity in reflecting drug safety, and the impact of oxaliplatin on DPLOs varied across different time points.

Furthermore, DEPs enrichment analysis was performed based on the proteomic results of different groups. When comparing the groups with and without synchronization treatment, enrichment of DEPs was observed across various pathways, including the rhythmic process pathway (Figure 5i). Additionally, the cell proliferation and cell killing pathways were enriched, which is consistent with the inhibitory effect of oxaliplatin^[^ ^52^, ^53^ ^]^ on DNA synthesis and cell proliferation (Figure 5i). When comparing the drug‐treated and untreated groups, we observed changes in multiple pathways after drug administration, with SS‐6 h group showing more pronounced alterations (Figure 5j; Figure S11, Supporting Information), including changes in the cell‐killing pathway. However, such changes were not observed in the SS‐18 h group. Oxaliplatin administered at 6 hours after synchronization (SS‐6h‐O) showed a more profound impact on DPLOs compared to 18 hours (SS‐18h‐O), implying time‐dependent variations in oxaliplatin toxicity (Figure 5j). Moreover, the number of enriched pathways between the control groups with and without oxaliplatin treatment (C‐6 h/C‐6h‐O) were minimal, possibly due to the lower sensitivity of non‐rhythmic DPLO in evaluating drug safety, as previous described (Figure 5j).

To evaluate the potential of Chronotoxici‐plate as an effective tool for assessing the efficacy of chronotherapy on those drugs, we selected liver‐related genes from the circadian‐related drugs among the Top 100 best‐selling drugs and generated a gene expression correlation heatmap (Table S1, Supporting Information). If a gene's expression varies over time in a rhythmic manner, its relative expression compared to the non‐rhythmic state can be represented by a periodic function symmetric about the x‐axis. Consequently, if the ratio of relative expression between two consecutive time points spaced over 6 hours was positive, then the next 6‐hour interval would be negative, and vice versa. The ratio between time points spaced 12 hours apart was always negative. The heatmap reveals that these genes exhibit periodic changes in rhythmic DPLO (Figure 5k), providing a foundation for exploring the optimal administration time and mechanisms of the aforementioned prevalent drugs. Furthermore, this approach also holds the promise for effective toxicity screening of drug candidates.

## 3 Conclusion

The crucial role of circadian biology in amplifying drug efficacy and minimizing toxicity is becoming increasingly acknowledged. The biological state of target cells is instrumental in modulating an organ's response to a drug at specific times, thereby affecting pharmacodynamics.^[^ ^1^ ^]^ Overlooking these time‐dependent effects may lead to premature dismissal of potentially useful drugs^[^ ^23^ ^]^ or provoke unnecessary side effects in patients.^[^ ^14^ ^]^

Highlighting the significance of considering circadian rhythms, the case of oxaliplatin is instructive. Initially, Rhone‐Poulenc‐Rorer^[^ ^54^ ^]^ in France halted Phase I clinical trials due to excessive toxicity. However, the drug found a new lease of life when Debiopharm, a Swiss company, focused on assessing its safety and efficacy using chronopharmacology. Following successful clinical trials,^[^ ^49^, ^55^, ^56^, ^57^ ^]^ oxaliplatin is now globally recognized as a vital treatment for cancer. This underscores the necessity for incorporating circadian rhythms into clinical trials and the development of novel therapies.

The comprehensive screening of drug administration timings could have a significant impact on the pharmaceutical industry, especially when considering drugs that have previously failed in clinical trials. Integrating chronotherapy considerations into the process of new drug development presents a unique opportunity for advancing medical treatment. However, developing high‐throughput and cost‐effective models that accurately represent the effect of the circadian clock on drug efficacy remains a significant challenge.

To tackle this challenge, we utilized 3D organoid printing technology to establish Chronotoxici‐plate, which incorporated in vitro synchronization treatment on DPLOs. This innovative tool enables circadian drug screening within a mere 4‐day period and comprehensive safety assessments within a week (Figure 3, 4). It represents a high‐throughput solution for studying drug administration in the context of circadian rhythms, facilitating a more rapid and accurate drug screening process. To simplify the incorporation of circadian assessments in clinical therapy, Cry1 has been identified as a potential companion diagnostic marker.

The culture of primary cells in vitro presents unique challenges due to the limited cell interactions in a 2D environment. These limitations lead to lower cell proliferation, diminished viability, and weakened self‐assembling abilities. In particular, the reduced interactions among hepatocytes result in hampered formation of bile canaliculi and a decrease in essential signaling pathways necessary for standard hepatocyte function.^[^ ^58^ ^]^

Our study, however, revealed that circadian organoids exhibit certain variations in biological activity compared to their non‐circadian counterparts (Figures 2, 4). These results indicate that the incorporation of circadian rhythmicity into primary organoid culture could provide promising avenues for enhancing organoid development. Future research will seek to illuminate the underlying mechanisms that contribute to the beneficial effects of rhythmicity on organoid development.

Further, our evaluation of oxaliplatin's safety using DPLOs in the Chronotoxici‐plate demonstrated its usefulness as a sensitive tool for assessing drug safety in the realm of chronopharmacology. The observation of a ten‐fold difference in IC50 values at different administration times aligns with the conclusion of clinical trials^[^ ^23^ ^]^ indicating varying toxicity levels of oxaliplatin depending on the time of administration (Figure 5d). Several studies have demonstrated the influence of circadian rhythms on the pharmacological effects of oxaliplatin, but few have investigated the underlying mechanisms of this rhythm and its interaction with oxaliplatin within mammalian organism.^[^ ^59^, ^60^, ^61^, ^62^ ^]^ The relationship between these two may be a breakthrough in the mechanism of action of oxaliplatin and deserves more in‐depth study.

The study also revealed the rhythmicity of popular drug targets in the Chronotoxici‐plate (Figure 5j), highlighting the potential to develop chronotherapy for existing drugs. Moreover, 3D organoid printing technology enables automated and high‐throughput organoid production and patterning in multi‐well plates. Compared to traditional animal models, DPLOs in Chronotoxici‐plate could offer over a 1000‐fold increase in chronotherapy screening efficiency, which supports the completion of chronotoxicity assessment within 7 days.^[^ ^63^, ^64^, ^65^, ^66^, ^67^, ^68^ ^]^

Despite these advancements, there are still several challenges and future research directions that require further exploration. First, it would be invaluable to expand the application of Chronotoxici‐plate to organoids derived from a variety of tissue types – such as kidney, heart, or even tumor tissues – would be invaluable. This could provide a means for conducting organ‐specific chronotherapy evaluations and exploring the pan‐organ applicability of *Cry1* as a companion diagnostic marker.

Second, recognizing the nuanced differences in circadian behaviors between rodents and humans – such as the polyphasic sleep patterns in rodents^[^ ^69^ ^]^ – prioritizing organoids derived from human cell sources, such as induced pluripotent stem cells (iPSCs) or primary human tissues, should be considered. This strategy would serve to enhance the clinical translational potential of Chronotoxici‐plate.

Finally, there are numerous commonly used drugs with significant chronotherapeutic potential that have not yet been evaluated for optimal timing of administration. This highlights the need for more comprehensive examinations of pharmaceuticals in future studies, further emphasizing the importance of this developing field.

## 4 Methods

### 4.1 Cell Culture

AML12 (alpha mouse liver 12) cells were used as the control of liver organoids. Cells were cultured in a medium comprising Dulbecco's modified Eagle's medium (DMEM/F12) (Gibco) supplemented with 10% fetal bovine serum (FBS) (Gibco), 1% (w/v) Penicillin‐Streptomycin‐Amphotericin B Solution (P/S/A) (Procell), 1× Insulin‐Transferrin‐Selenium (ITS) (Thermo Fisher), and 40 ng ml^−1^ dexamethasone (Glpbio).

Primary mouse liver tissues were obtained from BALB/c mice (male, 6–8 weeks old) euthanized by cervical dislocation. The tissue processing method was previously described^[^ ^74^ ^]^ The cells were then resuspended in DMEM/F12 supplemented with 10% (w/v) FBS and 1% (w/v) P/S/A. The cell number was counted for organoid fabrication.

### 4.2 DPLO Precursor Fabrication and Chronotoxici‐Plate Construction

The cells isolated from mouse liver were suspended in Matrigel (Corning) on ice to avoid the crosslink of Matrigel and the cell‐Matrigel mixture was loaded in a 1 mL syringe and installed in an injection pump. A fluorocarbon oil (HFE‐7000, 3 M Novec) was loaded into a 10 mL injection syringe and installed in another injection pump. Both pumps were placed in a 0–4 °C refrigerator. The two phases were co‐injected simultaneously through polytetrafluoroethylene (PTFE) tubing (the inner diameter ID = 560 µm, Woer) into a third piece of PTFE tubing via a 3‐way hand‐made PDMS connector. The Matrigel phase was injected at the flow rate of 8.0 µL mi^−1^n and subsequently sheared into monodisperse droplets by the fluorocarbon oil at the flow rate of 48.0 µL min^−1^.^[^ ^75^, ^76^ ^]^ The tubing, along with the Matrigel droplets collected in it was heated to 37 °C for 25 min to complete solidification. The tubing was then connected to a 3D printing device. On one end, the tubing‐in‐the‐printer was linked to a pump filled with oil, while the other end was connected to a three‐port connector equipped with an optical sensor. One outlet of this connector was connected to a controlled compressed gas port, and the other was linked to the printing nozzle. When the optical sensor at the nozzle identified an organoid in transit, the three‐port connector's compressed air valve was actuated, propelling the organoids into the designated 96‐well culture plate beneath, containing primary liver organoid culture medium^[^ ^29^ ^]^ (DMEM/F12 supplemented with 10% (w/v) FBS, 1 mM nicotinamide, 0.1 µM dexamethasone, 1% P/S/A, 1× ITS, 5 ng mL^−1^ EGF and 50 ng ml^−1^ HGF) individually (Figure 1). All organoid precursors were cultured at 37 °C in an incubator, supplied with 5% (v/v) CO2. Medium was changed every 2 days. Subsequently, the organoids were either harvested for further analysis or conditioned with drugs.

### 4.3 Non‐Engineered Organoids (NEOs) Fabrication

The cells isolated from mouse liver were suspended in Matrigel (Corning) on ice to avoid the crosslink of Matrigel and aliquotes of cell suspension were placed in a 96‐well plate format at 20 µl drop^−1^ so that a small dome of Matrigel formed in the center of each well, and plates were then inverted incubated at 37 °C for 25 min until the Matrigel solidified. Subsequently, primary liver organoid culture medium was added to each well. The culture medium was changed every 48 h. Afterward, the organoids were either harvested for further analysis or conditioned with drugs.

### 4.4 Serum Shock

The organoid reached a stable condition after ≈4 days. Then the primary liver organoid culture medium was exchanged with serum‐rich medium (DMEM/F12 supplemented with 50% FBS^[^ ^10^ ^]^), after 30 minitues,^[^ ^77^ ^]^ the medium was replaced with the aforementioned culture medium.

### 4.5 Cell Viability Assay

The viability of cells in the organoids or organoids precursor was assessed using a LIVE/DEAD staining kit (Yeasen, China). The organoids were washed with 1 × PBS buffer and then immersed with 2.0 × 10^−6^ M Calcein AM (the live cell staining dye) and 4.5 × 10^−6^ M PI (the dead cell staining dye) for 15 min, followed by gentle 1×PBS buffer washing. All microscopic images were captured under a confocal microscope (N‐STORM). All images were generated using z‐plot analysis in ImageJ.

The viability of cells in organoids was also evaluated using CellTiter‐Glo® 2.0 Cell Viability Assay by quantitating the amount of ATP present, which indicates the presence of metabolically active cells. Following removal of the culture medium, the organoids were washed with 1×PBS buffer and then immersed with CellTiter‐Glo® 2.0 Reagent. Finally, the luminescence value was measured by Spark multimode microplate reader (TECAN, SPARK).

### 4.6 Measurement of RNA concentration and real‐time quantitative PCR (RT‐qPCR)

Total RNA from organoids or 2D cells was extracted using Eastep® super total RNA extraction kit (Promega) and transcribed to cDNA by the PrimeScript™ RT reagent Kit (TAKARA). cDNA was used for qPCR using the GoTaq® qPCR Master Mix (Promega, USA) with RT‐qPCR instrument (Bio‐Rad, CA). The primers used for the qPCR reaction are shown in Table S1 (Supporting Information). To ensure uniform baseline values across all curves, each data point was normalized by the value at t = 0. This normalization eliminated variations attributed to distinct RNA extraction amounts, facilitating comparisons between different groups. Additionally, we evaluated the statistical significance (p‐value) of the curve fits were evaluated for both SS‐treated and control (C) groups.

### 4.7 Western Blot

Cells cultured in Matrigel were collected and lysed with RIPA buffer (MCE) containing protease and phosphatase inhibitors (MCE). Protein concentrations were determined by the BCA Protein Assay Kit (23 227, Thermo Fisher Scientific). Western blotting was conducted according to a standard protocol. Briefly, the proteins were loaded on 10% (w/v) SDS‐polyacrylamide gels and electrophoretically transferred to a PVDF membrane (Millipore). The primary antibodies were used as follows: β‐actin (1:1000, Cell Signaling Technology, 8H10D10), CRY1 (1:1000, Proteintech, 13474‐1‐AP). Detection was performed using a Chemiluminescent Western Blot detection kit (4AW012‐1000, 4A Biotech). The western blot results were analyzed using ImageJ (version 1.52).

### 4.8 Drug Safety Evaluation on DPLOs

Primary liver organoids were printed on 96‐well plates or 24‐well plates (for iTRAQ analysis). After 4‐day development, organoids began to be exposed to 0, 0.1, 0.01, 0.001, 0.0001 mM oxaliplatin for 36 h at different time points. Cell viability assay and other evaluating was performed as described above.

### 4.9 Measurement of Glutamic‐Oxalacetic Transaminase/ Glutamic‐Pyruvic Transaminase (GOT/GPT) Enzyme Activities

Organoids were exposed to 0.1 mM oxaliplatin for 36 h at designated time points (12 h,18 h) after SS treatment. After being collected and washed for 3 times with PBS, the organoids were suspended in cold physiological saline, and later were subsequently disrupted via ultrasonic cell homogenizer (Scientz‐IID) to extract lysed proteins. The activities of GOT and GPT were measured using detection kits purchased from Jiancheng Bioengineering Institute (Nanjing, China). The process followed the manufacturer's instructions. Optical density (OD) values were measured using an enzyme‐labeled instrument (TECAN, SPARK).

### 4.10 Histologic Analysis and Immunostaining and of Rhythmic DPLOs

DPLOs were cultured in the primary liver organoid culture medium for 4 days and subjected to SS. Subsequently, the DPLOs were fixed using a paraformaldehyde‐glutaraldehyde fixative (0.5%/0.625%, Biosharp) for 15 minutes at room temperature, followed by three‐time washes with PBS. Dehydration of the DPLOs was performed using a 20% saccharose solution. The samples were then immersed in an optimal cutting temperature (OCT) compound and incubated for 12 hours, after which they were rapidly frozen in liquid nitrogen. The samples were cryo‐sectioned into 10‐µm slides. HE staining was conducted following the manufacturer's instructions, and images were captured using an Olympus VS200 Research Slide Scanner.

For immunofluorescence, the slides were blocked with 10% (w/v) normal donkey serum in PBS supplemented with 0.1‐1% Triton X‐100 for 1 hour at room temperature. Subsequently, the slides were incubated at 4 °C for 12 hours with mouse Cytokeratin 19 (1:100, XH3643123, Invitrogen), rabbit Albumin (1:100, 0806–9, Huabio), mouse Cytokeratin 18 (1:100, M0407‐19, Huabio), rabbit HNF4a (A20865, 1:100, ABclonal), rat anti‐CD68 (1:100, 14‐0681‐82, Invitrogen). Slides were washed three times with PBS and then incubated with secondary antibodies, including anti‐rabbit secondary antibody (1:500) (Alexa Fluor 647, Abcam), anti‐mouse secondary antibody (1:500) (Alexa Fluor 488, Abcam), and anti‐rat secondary antibody (1:500) (Alexa Fluor 488, Abcam), at room temperature for 1 hour. Afterward, the slides were mounted using DAPI (1:1000), and the images were captured using a confocal microscope (N‐STORM). Negative control experiments were conducted in parallel.

### 4.11 Oil Red O Staining of DPLOs

Oil Red O staining was applied to evaluate the de novo lipogenesis in DPLOs samples. The samples were divided into 2 groups, one was cultured in the primary liver culture medium for 3 days, followed by subsequent collection. In contrast, the second group was cultured for an additional day, after which it underwent SS treatment or kept as control, and continued to be cultured until day 7. Subsequently, the DPLOs were fixed using a paraformaldehyde‐glutaraldehyde fixative (0.5%/0.625%, Biosharp) for 15 minutes at room temperature, followed by three‐time washes with PBS. Dehydration of the DPLOs was performed using a 20% saccharose solution. The samples were then immersed in an optimal cutting temperature (OCT) compound and incubated for 12 hours, after which they were rapidly frozen in liquid nitrogen. The samples were cryo‐sectioned into 10‐µm slides. Oil Red O staining (G1261, Solarbio) was conducted following the manufacturer's instructions, and images were captured using an Olympus VS200 Research Slide Scanner.

### 4.12 RNA‐Seq Analysis

The mRNA sequencing of the control group and SS groups was performed in BGI‐Shenzhen, China. Briefly, the control group was taken out after four‐day development and treated with the normal primary liver organoid culture medium, while the SS groups were treated using SS medium for 0.5 h in 37 °C and then cultured in the normal culture medium before being taken out at specific time points. The sequencing was performed on BGISEQ platform and SOAPnuke v1.5.2^[^ ^78^ ^]^ was used to filter out the low‐quality reads. And HISAT v2.0.4.^[^ ^79^ ^]^ was used to the alignment of reads to the reference genome GCF_0 00001635.26_GRCm38.p6.

### 4.13 iTRAQ Quantification Proteomics Analysis

The protein quantification of control group and SS group with or without oxaliplatin at different time points was performed with Isobaric tags for relative and absolute quantitation (iTRAQ) technology in BGI‐Shenzhen, China. Totally 906 357 spectrums were generated, 35 136 peptides and 5879 proteins were identified with 1% FDR. The quantification process followed the company's protocol.

### 4.14 Statistical Analysis

All the statistical analysis was performed using Origin 2021b (OriginLab Co, Northampton, MA). In measuring the RNA concentration of RT‐qPCR, we normalized each data point by the value at t = 0. A statistically significant difference was considered when the value of p < 0.05 (* indicates p < 0.05, ∗∗ indicates p < 0.01, ***indicates p < 0.001), using unpaired two‐failed Student's t‐test (comparing two experimental groups). Error bars in all figures are the standard deviations obtained from at least three independent measurements unless otherwise stated. All representative fluorescence images, H&E staining, and bright field images shown in the main text and supplementary information were independently repeated at least three times.

### 4.15 Ethics Statement

All the animal procedures involved in the study were reviewed and approved by the Animal Experimentation Ethics Committee at Tsinghua Shenzhen International Graduate School, Tsinghua University (Project 2023/22).

## Conflict of Interest

The authors declare no conflict of interest.

## Author Contributions

J.Z., Y.‐C.H., and W.W. contributed equally to this work. J.Z., J.L. and S.M. raised the innovation, designed the experiments, data analysis and organized the experiments. J.Z., W.W., Y.H., J.L., Z.Y., H.Y., Z.W and Y.Z performed the experimental work. J.Z., W.W., Y.H. and K.H. contributed to Figure preparation. S.M., J.Z., J.L. contributed to paper writing and revising. S.M. and J.L. conceived and advised on this work. All authors contributed to discussion.

## Supporting information

Supporting Information

## Data Availability

The data that support the findings of this study are available from the corresponding author upon reasonable request.
