# Supplementary material for: Chronotoxici‐Plate Containing Droplet‐Engineered Rhythmic Liver Organoids for Drug Toxicity Evaluation
Source: Adv Sci (Weinh). 2024 May 8;11(28):2305925. doi: 10.1002/advs.202305925 (PMC11267367; doi:10.1002/advs.202305925)
Supplement: Supplementary file 1 — Supporting Information [file ADVS-11-2305925-s001.pdf]

ADVANCED SCIENCE
Open Access

Supporting Information

for *Adv. Sci.*, DOI 10.1002/advs.202305925

Chronotoxici-Plate Containing Droplet-Engineered Rhythmic Liver Organoids for Drug Toxicity Evaluation

*Jiaqi Zhou, Yi-chun Huang, Wanlong Wang, Jiawei Li, Yibo Hou, Ziqi Yi, Haowei Yang, Keer Hu, Yu Zhu, Zitian Wang and Shaohua Ma**

Supplementary Material

Supplementary Table 1. Drugs of the top-100 best-seller list that target circadian genes and related to liver.

Rank	Sales, $	Trade name	Indications	Circadian-gene targets	Organs in which targets oscillate
11	794 m	Rituxan	Rheumatoid arthritis, Non-Hodgkin's lymp...	Fcgr2b, Fcgr3	L, K, S
20	538 m	Diovan	Hypertension, Heart failure	Agtr1a, Slco1b2,	H, AG, L, K, S
32	392 m	Tamiflu	Influenza	Neu1, Ces1g,	Lu, L, BF, K, C
38	346 m	Lidoderm	Pain	Egfr, Abcb1a	Lu, H, AG, BF, L,...
49	274 m	Alimta	Mesothelioma, Nonsmall cell lung cancer	Slc29a1	Lu, H, BS, BF, L,...
62	240 m	Janumet	Diabetes mellitus T2	Slc47a1,	H, BS, AG, Hy, L,...
66	236 m	Toprol XL	Hypertension, Heart failure	Abcb1a	Lu, H, AG, BF, L,...
78	209 m	Aciphex	Gastritis, GERD, Esophagitis	Abcg2	Lu, H, BS, WF, L,...

Rank and sales are based on USA 2013 Q1 data from Drugs.com. AG, adrenal gland; BF, brown fat; BS, brainstem; C, cerebellum; H, heart; Hy, hypothalamus; K, kidney; L, liver; Lu, lung; S, skeletal muscle; WF, white fat(*1*)

Supplementary Table 2. Primers for RT-qPCR reaction

Gene Name	Forward primer sequence	Reverse primer sequence
Hgf	ACCTACAGGAAAACTACTGTCG	TGCATTCAACTTCTGAACACTG
Fgf7	TGCATTCAACTTCTGAACACTG	TCCATGATGTTGTAGCTGTTCT
Tgfb1	CCAGATCCTGTCCAAACTAAGG	CTCTTTAGCATAGTAGTCCGCT
Gpt	CTTCAAGCAGTTTCAAGCAGAG	TTGAGGGAAGGAATACATAGCG
Got1	ACAAGAACACACCAATCTACGT	ATAGGGCCGAATGTCCTTAAAA
Got2	AGCCTTCAAGAGAGATACCAAC	CAGAAGCCTTACAGAATTCAGC
Cry1	CAGACTCTCGTCAGCAAGATG	CAAACGTGTAAGTGCCTCAGT
Gapdh	TGCACCACCAACTGCTTAGC	ACAGTCTTCTGGGTGGCAGTG

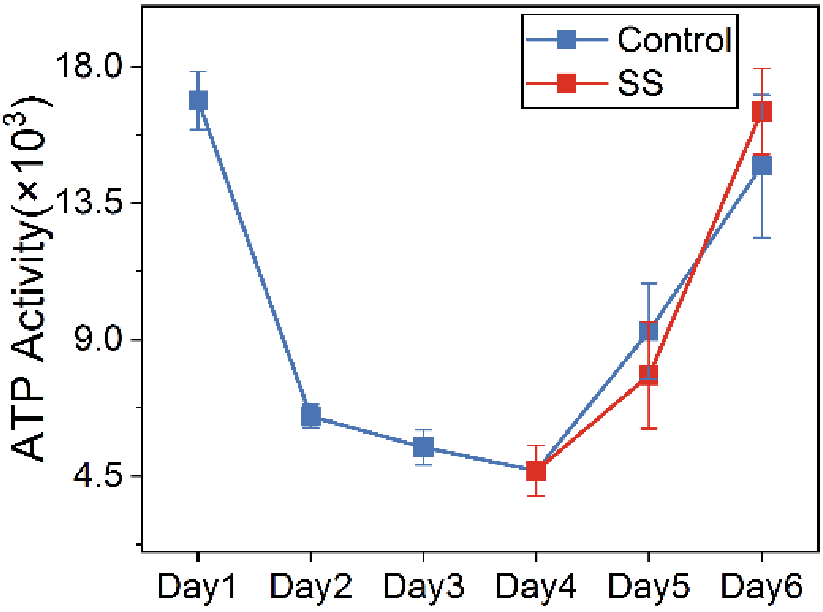

Supplementary Figure 1. Assessment of cell viability in DPLOs over Day 1 to Day 4, followed by a comparison between the SS group (SS, indicated by the red line) and the control group (C, represented by the blue line) on Day 5 and Day 6.

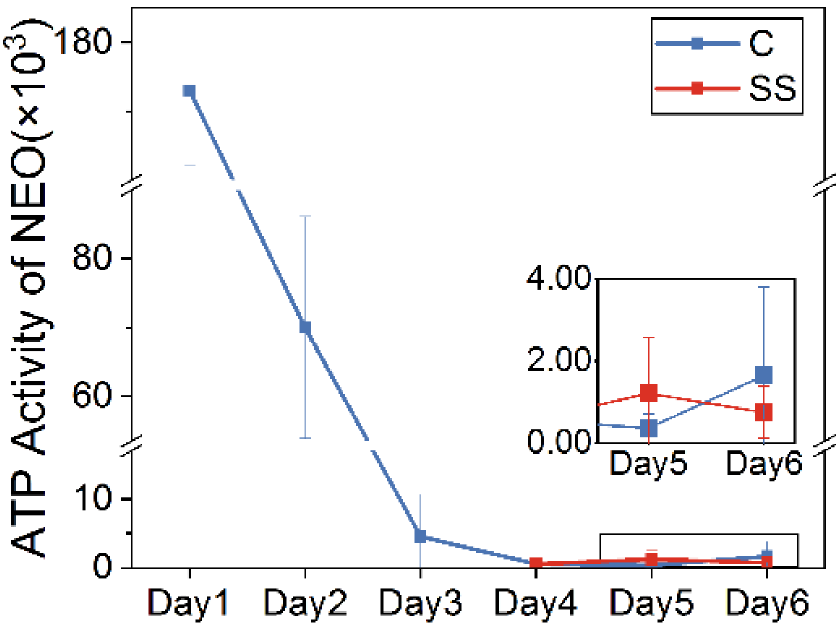

Supplementary Figure 2. Assessment of cell viability in NEO over Day 1 to Day 4, followed by a comparison between the SS group (SS, indicated by the red line) and the control group (C, represented by the blue line) on Day 5 and Day 6.

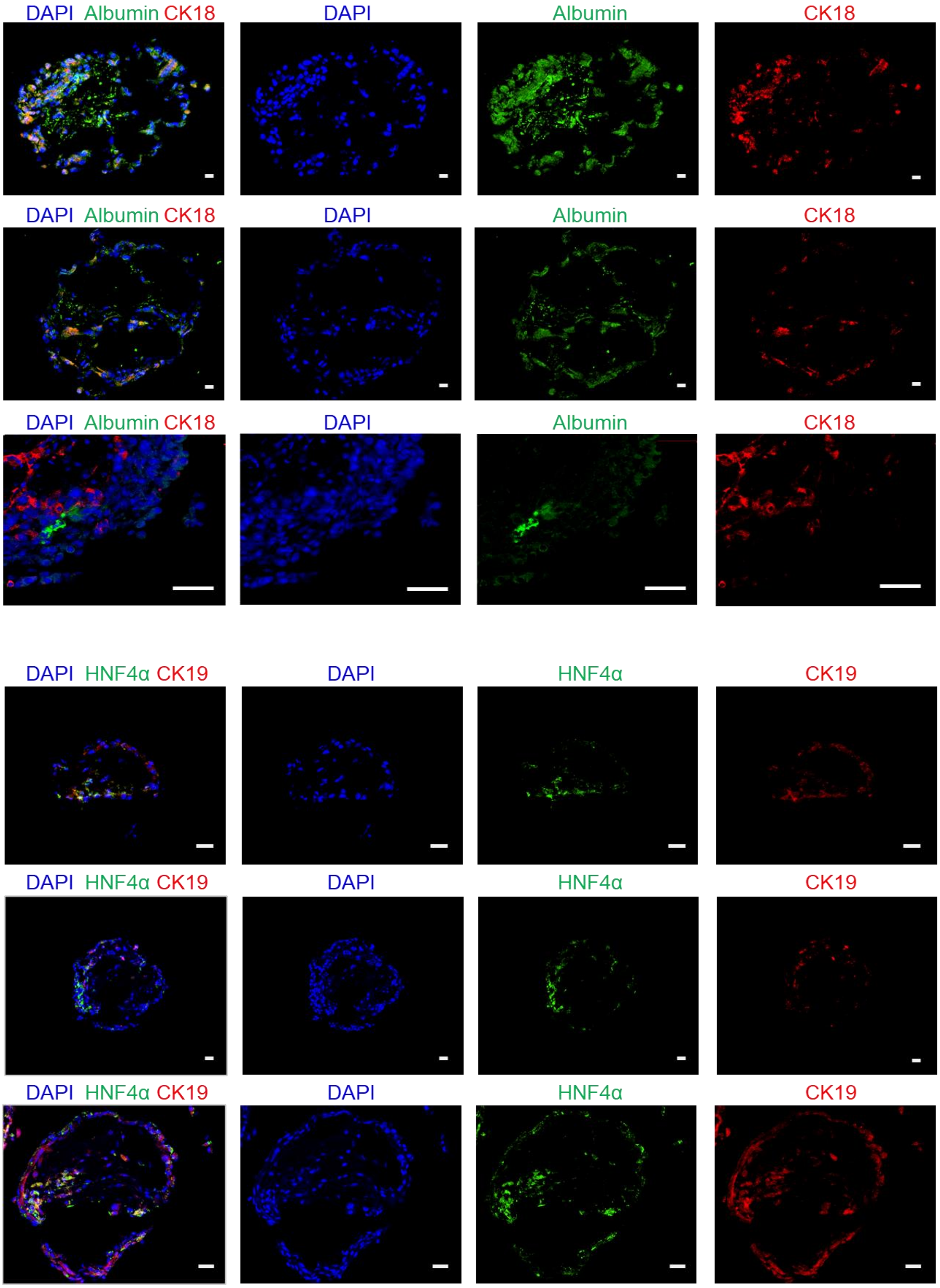

Supplementary Figure 3. Confocal images of a cryo-sectioned DPLO co-stained for HNF4a

(green), CK19 (red), albumin (green), CK18 (red), and DAPI (blue). Scale bar = 50 μm.

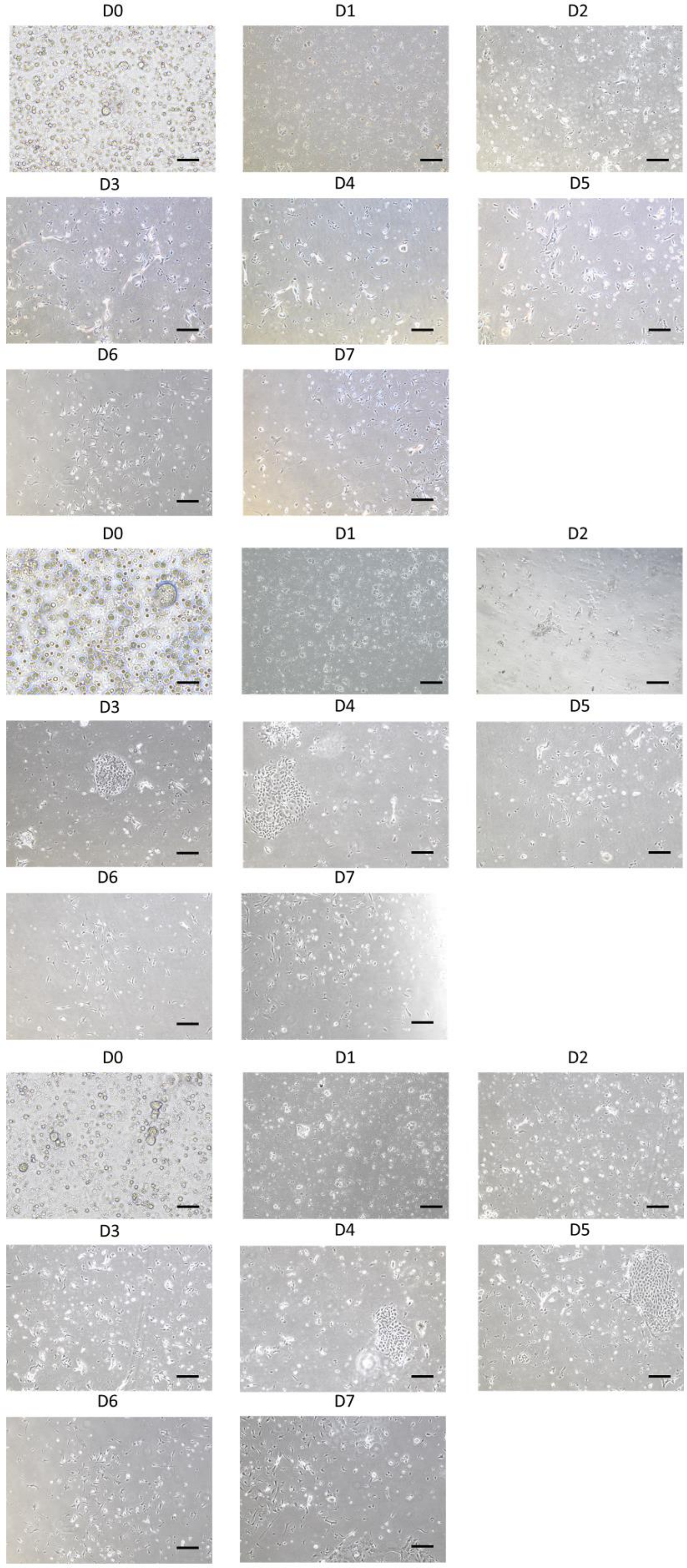

Supplementary Figure 4. Observation of mouse primary liver cells in 2D culture under bright-field microscopy during Day 0 to Day 7. Scale bar = 500 μm.

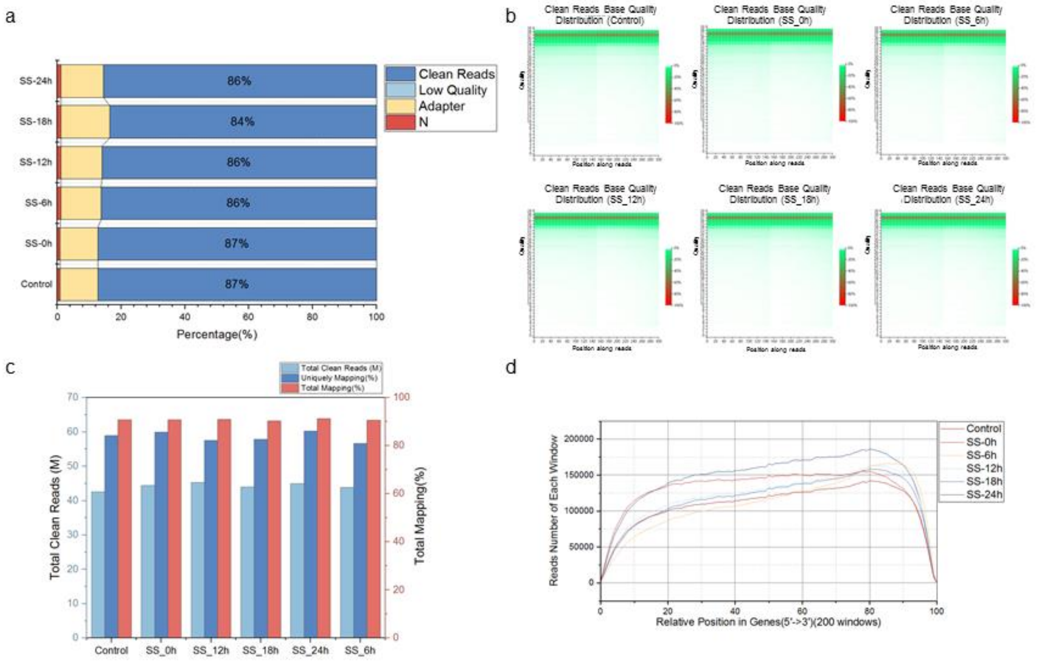

Supplementary Figure 5. mRNA sequencing quality control. a. Proportions of different nucleotide types in each sample as determined by mRNA sequencing. N: reads with an unknown nucleotide content exceeding 5% of the total raw reads. Adapter: reads contaminated with adapters. Low Quality: reads with a base quality below 15 account for more than 20% of the total bases in the read. Clean Reads: filtered reads after quality control. The high percentage of clean reads (>80%) indicates high-quality composition. b. Base quality distribution of clean reads in each sample as determined by mRNA sequencing. The X-axis represents the position of bases in the reads, and the Y-axis represents the base quality value. Each point on the graph represents the total number of bases at that position with a specific quality value, with darker colors indicating higher counts. The low proportion of low-quality bases (Quality < 20) indicates good sequencing quality. c. Mapping of sequencing fragments from each sample to the reference genome as determined by mRNA sequencing. After obtaining clean reads, HISAT was used to align them to the reference genome

sequence, demonstrating the absence of exogenous contamination and a high mapping rate. d. Distribution of reads on transcripts in each sample as determined by mRNA sequencing.

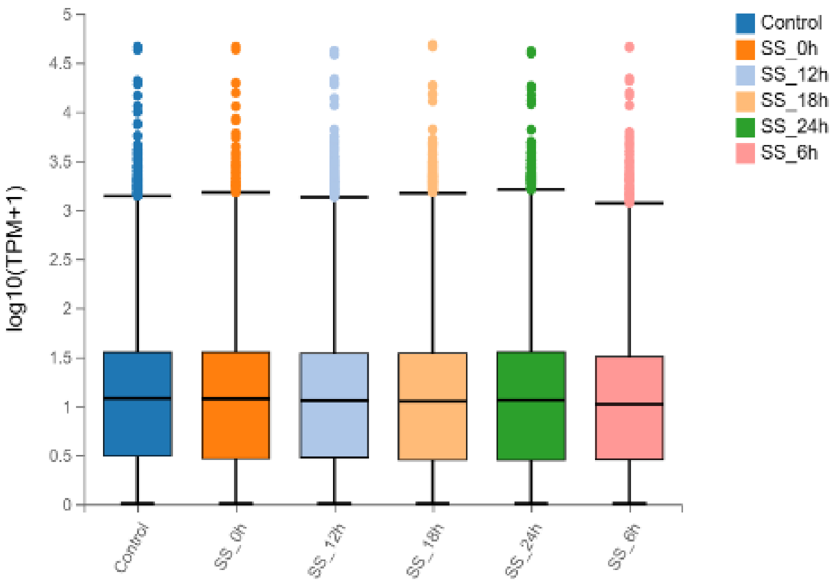

Supplementary Figure 6. Differential gene expression profiles among different samples as determined by mRNA sequencing.

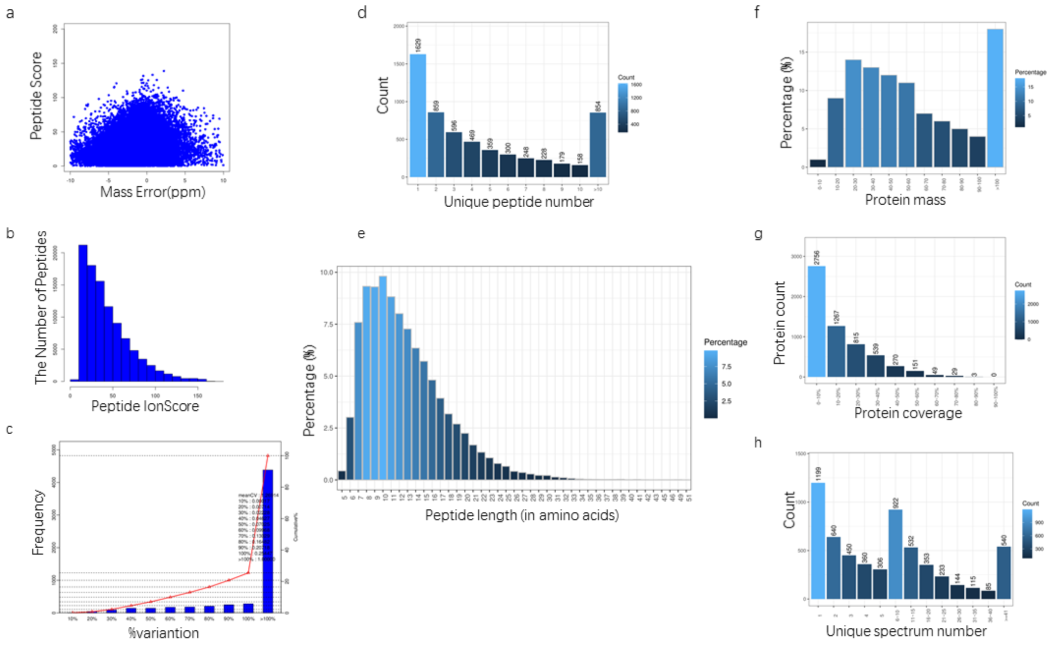

Supplementary Figure 7. Isobaric Tags for Relative and Absolute Quantitation (iTRAQ)

quality control. a. Peptide ion mass deviation distribution. The abscissa is the mass deviation between the theoretical mass-to-charge ratio of peptide ions and the mass-to-charge ratio measured by mass spectrometry experiments, and the unit is ppm, which is one millionth, which is a relative unit. The ordinate is the MASCOT peptide score. b. Peptide ion score distribution. The abscissa is the MASCOT peptide score; the main ordinate The Number of Peptides corresponds to the bar graph in the graph, indicating the number of identified peptides with the corresponding ion score. c. coefficient of variation (CV) distribution in replicate. X-axis is the deviation between the protein ratio of the repetitive samples. Y-axis is the percentage that protein at a certain angle comprises quantified protein amount. d. Unique peptide number distribution. The X-axis shows the unique peptide number of each protein and the Y-axis shows the corresponding protein number. The trend of this picture describes that most of the identified proteins contain less than 10 peptides and protein quantity becomes less with the increase of peptide. e. Peptide length distribution. This picture depicts the percentage of different peptide lengths. X-axis: the peptide length; Y-axis: the corresponding peptide percentage. f. Protein mass distribution. X-axis: Molecular weight(kDa); Y-axis: Percentage of protein number. g. Protein Coverage distribution. The X-axis shows the protein coverage range and the Y-axis shows the corresponding protein number. h. Unique spectra number distribution. The X-axis shows the unique spectrum number of each protein and the Y-axis shows the corresponding protein number.

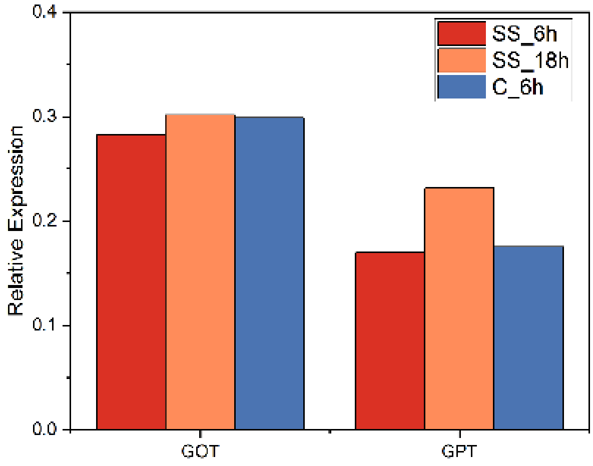

Supplementary Figure 8. GOT and GPT expression levels by iTRAQ analysis in DPLOs at various time points after SS, as well as in the control group.

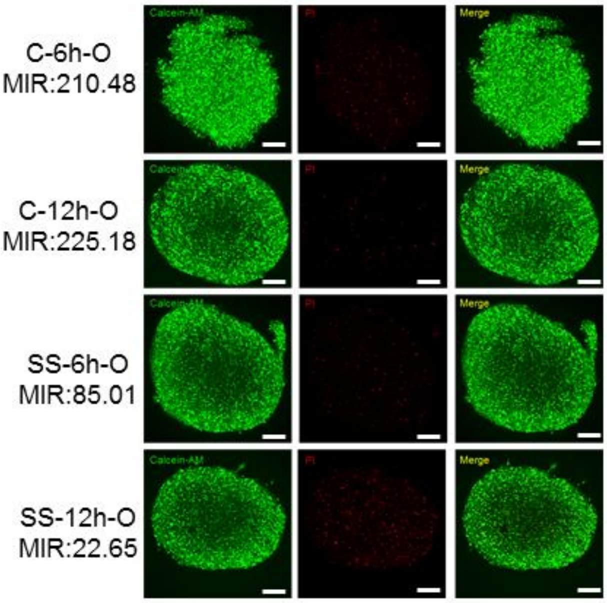

Supplementary Figure 9. Confocal microscopy image depicting the viability of DPLOs under 36-hour 10^{-4} mM oxaliplatin treatment beginning at the different time point after SS or control treatment through whole mount staining using Calcein-AM/PI live/dead staining. Scale bar = 100 µm.

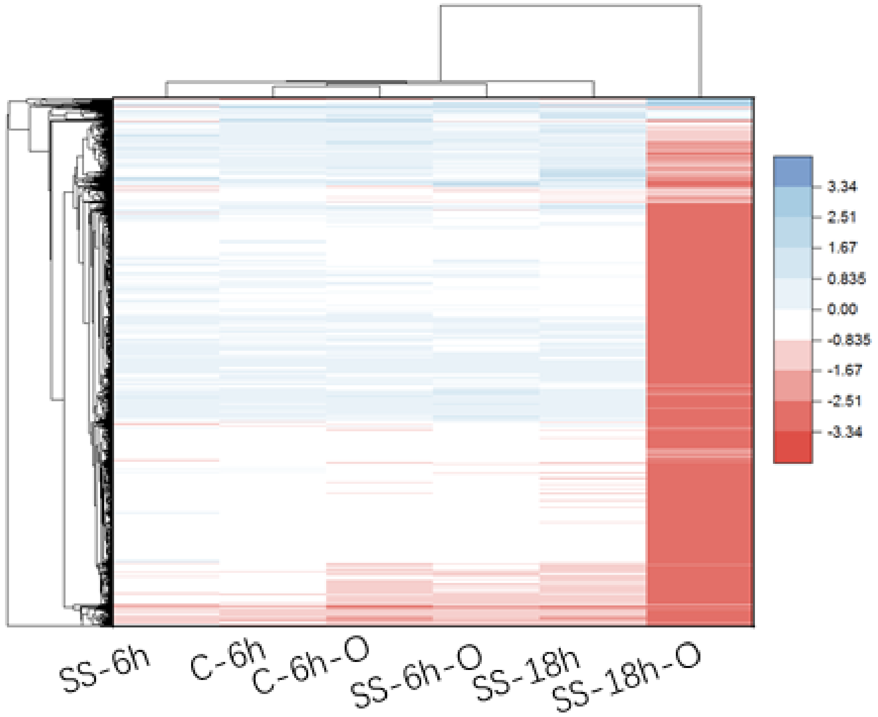

Supplementary Figure 10. Heatmap visualization of differentially expressed proteins identified by iTRAQ analysis in DPLOs subjected to 0.02mM oxaliplatin treatment for 36h or treated with same volume solvent at different time points following SS, as well as the control group.

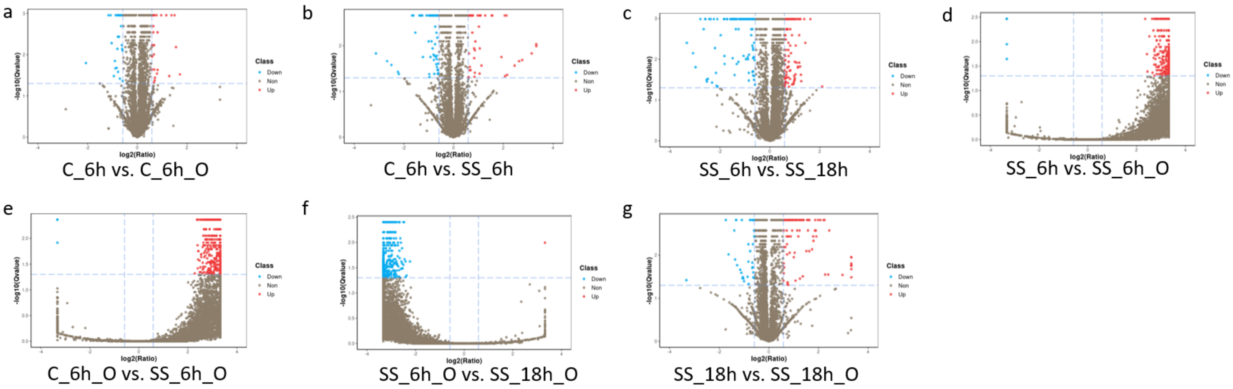

Supplementary Figure 11. The upregulation and downregulation of protein expression identified by iTRAQ analysis in DPLOs subjected to 0.02mM oxaliplatin treatment for 36 hours or treated with same volume solvent at different time points after SS compared to the control group. The x-axis represents the $\log_2$-fold change values, while the y-axis represents the $-\log_{10}$ of the significance values after transformation.

References

1. R. Zhang, N. F. Lahens, H. I. Ballance, M. E. Hughes, J. B. Hogenesch, A circadian gene expression atlas in mammals: implications for biology and medicine. Proc Natl Acad Sci U S A 111, 16219-16224 (2014).
